# Current News

**Published:** 1901-10

**Authors:** 


### Current News.

### NATIONAL ASSOCIATION OF DENTAL EXAMINERS.

At the last meeting of the National Association of Dental Examiners, held at Milwaukee on August 2 to 6, the following officers and committees were elected and appointed:

President, John F. Dowsley, 175 Tremont Street, Boston, Mass.; Vice-President, Chas. A. Meeker, 29 Fulton Street, Newark, N. J.; Second Vice-President, J. A. Hall, Collinsville, Ala.; Third Vice-President, B. L. Thorpe, LaClede and Vandewenter Avenues, St. Louis, Mo.; Secretary and Treasurer, J. Allen Osmun, 588 Broad Street, Newark, N. J.

*Committee on Colleges.*—C. C. Chittenden, Chairman, Madison, Wis.; J. A. Hall, Collinsville, Ala.; H. J. Burkhart, Batavia, N. Y.

*Committee on Conference.*—M. F. Finley, Chairman, Washington, D. C.; E. E. Kirkpatrick, Oklahoma; Chas. A. Meeker, Newark, N. J.

*Membership Committee.*—Geo. Everett Mitchell, Haverhill, Mass.; Melville A. Mason, Indianapolis, Ind.; Max. N. Ebele, Louisville, Ky.

*Committee on Contracts and Arrangements.*—Chas. A. Meeker, Newark, N. J.

J. Allen Osmun,
*Secretary.*

### NATIONAL ASSOCIATION OF DENTAL FACULTIES.

At the annual meeting of the National Association of Dental Faculties, held at Milwaukee, August 2 to 6, 1901, the following officers were elected for the ensuing year:

President, Dr. Wilbur F. Litch, Philadelphia; Vice-President, Dr. G. V. I. Brown, Milwaukee; Secretary, Dr. J. H. Kennerly, St. Louis; Treasurer, Dr. W. H. Morgan, Nashville, Tenn.

*Executive Committee.*—Drs. H. B. Tileston, Louisville, Ky.; S. W. Foster, Atlanta, Ga.; D. J. McMillan, Kansas City; J. B. Willmott, Toronto, Canada.

*Committee on Law.*—Drs. H. W. Morgan, Nashville; W. M. Montell, Baltimore; F. D. Weisse, New York.

*Ad Interim Committee.*—Drs. J. P. Gray, Nashville; W. T. McLean, Cincinnati; A. H. Peck, Chicago.

---

### HARVARD DENTAL ALUMNI ASSOCIATION.

THE thirtieth annual banquet was held at Boston, on the evening of June 24, 1901, with one hundred and twenty-three persons present.

The guest of the evening was General Curtis Guild, Jr., of Boston, who spoke upon "The Duties of a Liberal Education."

The following were elected officers for the ensuing year:

President, Henry W. Gillett, Newport, R. I.; Vice-President, Luther D. Shepard, Boston, Mass.; Secretary, Waldo E. Boardman, Boston, Mass.; Treasurer, Harry S. Parsons, Boston, Mass.

*Executive Committee.*—Waldo E. Boardman, Boston, Mass.; William P. Cooke, Boston, Mass.; Charles E. Perkins, Brockton, Mass.

WALDO E. BOARDMAN,
*Secretary.*

---

### DENTAL COMMISSIONERS OF CONNECTICUT.

THE following have been appointed as Dental Commissioners of Connecticut: Edward W. Pratt, President, East Hartford; Wm. H. Loomis, Rockville; W. E. Hyde, Danielson; Horace Bascom, New Haven; J. Tenney Barker, Recorder, Wallingford. There has been no change in our State law as reported.

J. TENNEY BARKER,
*Recorder.*

### NEW JERSEY STATE DENTAL SOCIETY.

At the annual meeting of the New Jersey State Dental Society at Asbury, Park, July 18, 1901, the following officers were elected for the ensuing year:

President, Wm. L. Fish, Newark; Vice-President, Frank L. Hindle, New Brunswick; Secretary, Chas. A. Meeker, Newark; Treasurer, Henry A. Hull, New Brunswick.

*Executive Committee.*—Frank L. Hindle, New Brunswick; A. S. Sutphin, Newark; A. Irwin, Camden; W. W. Hawke, Flemington; Oscar Adelberg, Elizabeth.

*Membership Committee.*—J. A. Duffield, Camden; G. M. Holden, Hackettstown; W. H. Pruden, Paterson; T. Star Dunning, Paterson; H. P. Marshall, Newark.

C. A. Meeker,
*Secretary.*

---

### NORTHEASTERN DENTAL ASSOCIATION.

The seventh annual meeting of the above Association is to convene in Springfield, Mass., October 30 and 31 and November 1, 1901. The committee promises original essayists of more than local reputation. Forty clinics,—table and chair. Memorial Building, where the meeting is to be held, has three floors,—upper for meeting, middle for exhibitors, lower for clinics. One and one-third fare, certificate plan, on the railroads.

Springfield is a well-located railroad centre, with eight first-class hotels. All ethical dentists are invited.

Edgar O. Kinsman,

*Secretary.*

Cambridge, Mass.

---

### SOUTHERN CALIFORNIA DENTAL ASSOCIATION.

The fourth annual meeting of the Southern California Dental Association will be held in Los Angeles on October 8 and 9, 1901.

L. E. Ford,
*Secretary.*

### NATIONAL DENTAL ASSOCIATION.

The National Dental Association, at its meeting at Milwaukee, August 6, 1901, elected the following officers for the ensuing year:

President, J. A. Libbey, Pittsburg, Pa.; Vice-President, East, S. H. Guilford, Philadelphia, Pa.; Vice-President, West, Wm. P. Dickinson, Minneapolis, Minn.; Vice-President, South, L. G. Noel, Nashville, Tenn.; Recording Secretary, A. H. Peck, Chicago, Ill.; Corresponding Secretary, Josephine Pfeifer, Chicago, Ill.; Treasurer, Henry W. Morgan, Nashville, Tenn.

*Executive Council.*—H. J. Burkhart, B. Holly Smith, J. Y. Crawford, M. F. Finlay, C. C. Chittenden.

*Executive Committee.*—C. S. Butler, W. N. Cogan, G. V. I. Brown.

*Committee on State Organization.*—J. Taft, Jas. McManus, H. W. Morgan.

*Publication Committee.*—A. H. Peck, F. L. Gilmer, G. V. I. Brown.

*Local Committee of Arrangements.*—H. J. Burkhart, M. O. Cooley, C. W. Stainton.

Next place of meeting Niagara Falls, N. Y.

---

### UNION MEETING OF THE SIXTH, SEVENTH, AND EIGHTH DISTRICT DENTAL SOCIETIES, STATE OF NEW YORK.

The thirty-third Union Meeting of the above Societies will be held in the Assembly-Room of the Osburn House, Rochester, N. Y., Tuesday, Wednesday, and Thursday, October 29, 30, and 31, 1901.

### PRELIMINARY ANNOUNCEMENT.

"Will Nitrous Oxide and Oxygen supplant Ether and Chloroform in General Surgery and Nitrous Oxide alone in Dental Surgery?" Dr. W. J. Roe, Philadelphia.

"The Enamel of the Central Incisors." Dr. Sylvester Moyer, Galt, Ontario, Canada.

"Some Embarrassing Educational Problems." Dr. W. C. Barrett, Buffalo.

"Amalgam: The Place it has won in Dentistry." Dr. F. A. Balachey, Buffalo.

"The Gingival Border from a Scientific Stand-Point." Dr. S. B. Palmer, Syracuse.

"The Ethical Relation of Dentist to Patient." Dr. A. C. McAlpine, Warren, Pa.

"Some New Lights on the Etiology of Pyorrhœa Alveolaris." Dr. J. B. Ernsmere, Buffalo.

Subject to be announced. Dr. R. H. Hofheinz, Rochester.

"Surface Markings upon the Teeth." Dr. J. J. Madden, Buffalo.

Subject to be announced. Dr. J. N. Crouse, Chicago, Ill.

Subject to be announced. Dr. B. S. Hert, Rochester.

"Diagnosis and Treatment of Malocclusion." Illustrated with models and appliances. Dr. H. A. Pullen, Buffalo.

"Utilization of Artificial Light." Mr. E. L. Elliott, Newark, Ohio.

Subject to be announced. Mr. W. A. Purrington, New York.

The committee have under arrangement other important additions. There will be a great many clinics, together with a complete dental exhibit. The committee are making strenuous efforts to make this one of the best Union Meetings ever held by the Societies and well worthy of your attendance. Members of the profession are cordially invited.

DR. F. MESSERSCHMITT,
*Chairman.*

138 MAIN STREET, East, ROCHESTER, N. Y.

